# Fracture Epidemiology in Skateboarding vs. Snowboarding

**DOI:** 10.1177/19417381251353773

**Published:** 2025-07-31

**Authors:** Viktor Schmidt, Mats Wadsten, Anders Brüggemann, Yasmin D Hailer, Olof Wolf

**Affiliations:** † Department of Clinical Sciences at Danderyd Hospital, Karolinska Institutet, Stockholm, Sweden; ‡ Department of Surgical and Perioperative Sciences at Umeå University, Sweden; § Department of Surgical Sciences/Orthopaedics, Uppsala University, Uppsala, Sweden

**Keywords:** boarding sports, fractures, winter sports, trauma, epidemiology, high-energy trauma, snowboarding, skateboard, treatment

## Abstract

**Background:**

Boarding sports, such as skateboarding and snowboarding, are associated with a significant risk of fractures. This study provides a comprehensive overview of the epidemiology, fracture locations, and treatment approaches for skateboarding and snowboarding-related fractures using data from the Swedish Fracture Register.

**Purpose:**

To provide a comprehensive overview of the epidemiology, fracture locations, and treatment modalities for fractures incurred during skateboarding and snowboarding.

**Study Design:**

Descriptive epidemiology study.

**Level of Evidence:**

Level 4.

**Methods:**

This observational study included all patients registered in the Swedish Fracture Register who sustained fractures while snowboarding or skateboarding from January 2015 to December 2023. Variables studied were age, sex, trauma energy level, seasonal variation, fracture location (body part), and treatment modality.

**Results:**

A total of 5155 patients (28% women) with 5446 fractures were included. Adults (≥16 years old) comprised 58% of all patients. The cohort experienced an approximately equal number of fractures from skateboarding (55%) and snowboarding (45%). A greater propensity for high-energy trauma injuries was observed among snowboarders and males. The mean age was similar in both groups, slightly above 20 years. Upper extremity fractures were the most common in both sports. However, discrepancies were noted: lower extremity fractures were more common in skateboarders, whereas injuries to the spine and pelvis were about 8 times more common in snowboarders. Specific injury patterns, such as the “snowboarder’s fracture” and “skateboard elbow,” appear unique to each sport.

**Conclusion:**

While skateboarding and snowboarding share similarities, notable differences exist in lower extremity and spinal fractures. Furthermore, specific fracture patterns are characteristic of each sport. Understanding these differences is crucial for developing targeted prevention strategies and improving safety measures.

T he popularity of skateboarding and snowboarding has increased since the 1990s, leading to a rise in associated injuries.^10,16^ Whether on snowy slopes or paved roads, engaging in board sports is closely linked to a substantial risk of fractures. In adolescents, 60% of injuries sustained while skateboarding or snowboarding are fractures, almost double that of alpine skiing (35%), where knee sprains and ligament tears are the leading injury types.^5^

Skateboarding, which originated in the 1950s as an alternative to surfing, has evolved into multiple disciplines, including street skating, park skating, vert skating, and longboarding. Street and park skateboarders often perform technical tricks on ledges, stairs, and rails, whereas vert skaters specialize in aerial tricks on ramps. Longboarding, on the other hand, is primarily used for transportation or downhill racing at high speeds, with riders often experiencing falls at considerable velocities. These distinctions in skateboarding styles influence the types and locations of injuries sustained by participants.

Snowboarding is a relatively new sport, first emerging in the 1960s before gaining widespread popularity at the end of the 1990s when it was introduced as an Olympic event. Unlike skateboarding, which can be practiced in urban settings, snowboarding requires access to designated slopes, ski lifts, and specific weather conditions, making it a seasonal sport in most regions.

Sweden, with a population of approximately 10 million people, has a strong affinity for winter sports. In the winter of 2021-2022, approximately 2 million individuals engaged in snowboarding and skiing in Sweden, amounting to 10 million days on the slopes.^24^ Given the large number of individuals susceptible to potential harm, understanding injury patterns and identifying areas for enhanced safety measures becomes crucial.

Similar to other sports, snowboarding has its unique injury panorama.^20^ Because snowboarders share the slopes with participants of other alpine sports, comparisons between them are common.^1,7,12,25^ Snowboarders have been suggested to have a threefold higher injury rate than skiers.^14^ There are ongoing discussions concerning the applicability of helmet recommendations for snowboarding to its summer equivalent, skateboarding, particularly longboarding, which constitutes a distinct subset within the skateboarding culture.^8^ However, a direct comparison of the fracture patterns between skateboarders and snowboarders has yet to be thoroughly addressed. Given the similarities between these boarding sports and the riders who engage in them, it is plausible to assume that their fracture epidemiology may also be similar.

The wrist, shoulder, and lower leg are the primary areas prone to snowboarding injuries.^1,12^ Skateboarding injuries often affect these same anatomical areas.^8,18^ This study aims to use data from the Swedish Fracture Register (SFR) to provide a comprehensive overview of the epidemiology, fracture locations, and treatment modalities for fractures incurred during skateboarding and snowboarding within a large Swedish cohort.

## Methods

### Study Design and Setting

A population-based national cohort study was conducted using data from the SFR.^17^ The SFR was established in 2011 to gather data on orthopaedic fractures and treatment procedures to enhance fracture care. The register collects data on patient demographics, injury causes, fracture details, and subsequent treatments. Registrations are completed by the treating physician, and registration can be performed in a stepwise manner with facilities adding information on fracture and treatment if the patient is transferred for further care or follow-up. Fractures within the SFR are primarily categorized using the Arbeitsgemeinschaft für Osteosynthesefragen/Orthopaedic Trauma Association (AO/OTA) 2007 classification system.^15^ Since 2021, the register has had 100% coverage, with all orthopaedic departments in Sweden participating. The national completeness is estimated to be around 60% when compared with the National Patient Register, and multiple studies have confirmed the high accuracy and validity of its registrations.^2,11,19,28^

The Strengthening the Reporting of Observational Studies in Epidemiology (STROBE) guidelines for reporting observational studies were followed.^27^

### Patient Selection

Patients with fractures registered with the injury mechanisms “snowboarding injury” and “skateboarding injury” in the SFR between January 1, 2015, and December 31, 2023, were identified and extracted using injury cause coding (W02 and SFR subgroups).

### Outcome Variables

Demographic data collected included age, sex, type of injury (high-energy or low-energy trauma), fracture location (body part), fracture classification, associated fractures, and primary treatment modality. The energy level associated with the fracture cause was classified as high, low, or missing/unknown based on the registering physician’s estimation. Treatment was categorized as nonoperative or operative. A cutoff of <16 years of age was used to differentiate between adult and pediatric populations.

### Statistical Analysis

**Figure 1.**
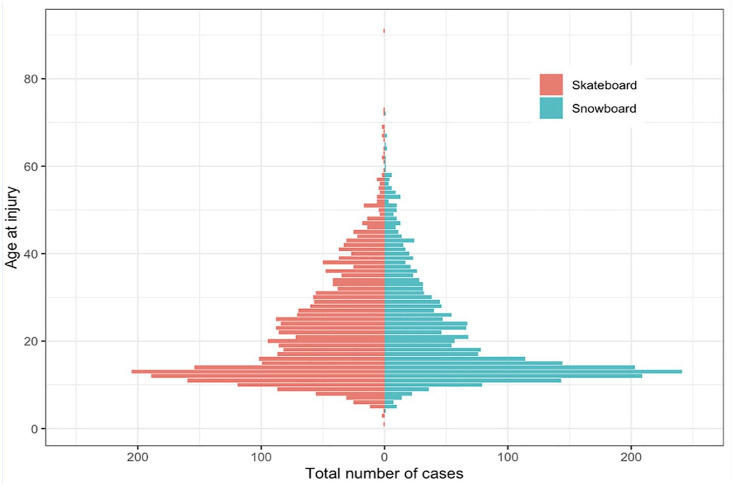
Bar plot illustrating the age distribution at injury for 5249 patients registered in the SFR and diagnosed with fractures resulting from skateboarding or snowboarding accidents.

Categorical variables are presented as frequencies and percentages, and continuous variables as means with standard deviations (SDs) or medians with interquartile ranges (IQRs). Age distribution by sport is illustrated using a bar plot (Figure 1). Fracture occurrence was analyzed for annual, monthly, and weekday variation. Descriptive statistical analyses and tables were computed using R version 4.0.2 (R Foundation for Statistical Computing, Vienna, Austria).

### Ethical Considerations

The study was approved by the Swedish Ethical Review Authority (DNR 2022-04355-01) and was conducted according to the ethical principles of the Helsinki Declaration, revised in 2013.

Data from the SFR are not publicly available due to Swedish legislation on public access and secrecy. The dataset can be retrieved from the Center of Registers, Västra Götaland, Sweden, after ethical approval from the Swedish Ethical Review Authority.

## Results

### Demographics

**Figure 2.**
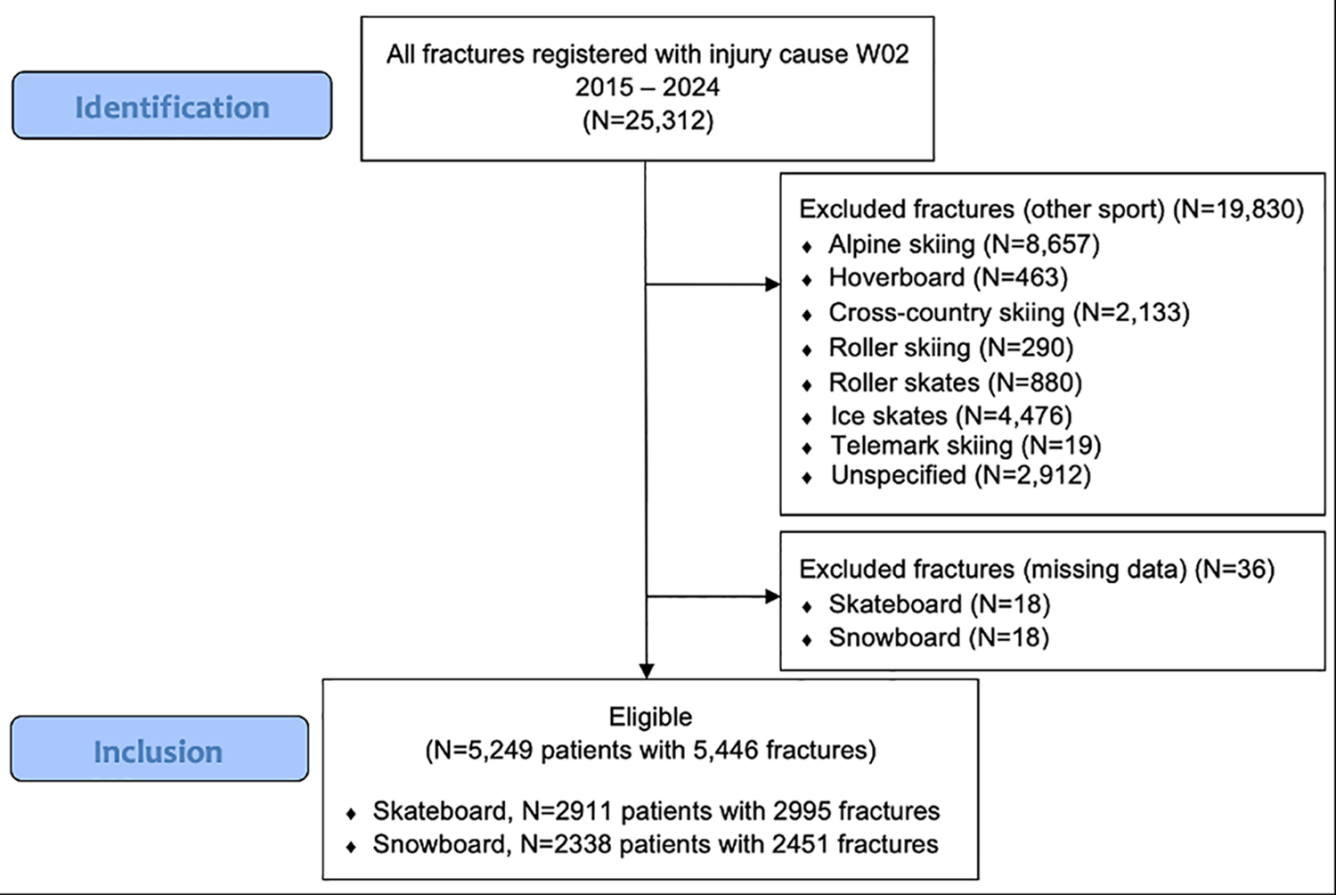
Flowchart of patients injured in snowboarding or skateboarding accidents in the SFR between January 2015 and December 2023.

The study included 5155 patients (28% women) with a total of 5446 fractures (Figure 2 and Supplementary Figure 1). The median age of skateboarders was 19 years (IQR 13-29), while snowboarders had a median age of 16 years (IQR 13-26) (Figure 2). Children comprised 42% of the cohort (n = 2156). Among skateboarders, 61% of patients were adults, compared with 54% among snowboarders (Table 1).

**Table 1.** Demographics and injury/fracture-related characteristics for 5155 patients sustaining a fracture from snowboarding or skateboarding in the Swedish Fracture Register from January 2015 to December 2023. Mean (SD) and median [range] for age and distribution (number (%)) for other variables. Patients aged ≥16 years at the time of injury were classified as adults

	Adult		Child		Overall	
	Skateboard (N = 1748)	Snowboard (N = 1251)	Skateboard (N = 1100)	Snowboard (N = 1056)	Skateboard (N = 2848)	Snowboard (N = 2307)
**Age**						
Mean (SD)	28.7 (10.2)	28.2 (10.7)	11.5 (2.37)	12.3 (2.03)	22.1 (11.7)	20.9 (11.2)
Median [min, max]	26.0 [16.0, 91.0]	25.0 [16.0, 72.0]	12.0 [1.00, 15.0]	13.0 [4.00, 15.0]	19.0 [1.00, 91.0]	16.0 [4.00, 72.0]
**Sex**						
Female	312 (17.8%)	437 (34.9%)	315 (28.6%)	397 (37.6%)	627 (22.0%)	834 (36.2%)
Male	1436 (82.2%)	814 (65.1%)	785 (71.4%)	659 (62.4%)	2221 (78.0%)	1473 (63.8%)
**Injured side**						
Right	809 (46.3%)	550 (44.0%)	474 (43.1%)	538 (50.9%)	1283 (45.0%)	1088 (47.2%)
Left	928 (53.1%)	631 (50.4%)	624 (56.7%)	513 (48.6%)	1552 (54.5%)	1144 (49.6%)
Unknown	11 (0.6%)	70 (5.6%)	2 (0.2%)	5 (0.5%)	13 (0.5%)	75 (3.3%)
**Energy level**						
High-energy	135 (7.7%)	193 (15.4%)	52 (4.7%)	131 (12.4%)	187 (6.6%)	324 (14.0%)
Low-energy	1263 (72.3%)	740 (59.2%)	798 (72.5%)	665 (63.0%)	2061 (72.4%)	1405 (60.9%)
Missing	186 (10.6%)	133 (10.6%)	152 (13.8%)	115 (10.9%)	338 (11.9%)	248 (10.7%)
Unknown	164 (9.4%)	185 (14.8%)	98 (8.9%)	145 (13.7%)	262 (9.2%)	330 (14.3%)
**Open fracture**						
No	1722 (98.5%)	1249 (99.8%)	1083 (98.5%)	1054 (99.8%)	2805 (98.5%)	2303 (99.8%)
Yes	26 (1.5%)	2 (0.2%)	17 (1.5%)	2 (0.2%)	43 (1.5%)	4 (0.2%)
**Associated**						
≥1 other fracture	58 (3.3%)	62 (5.0%)	18 (1.6%)	41 (3.9%)	76 (2.7%)	103 (4.5%)
None	1690 (96.7%)	1189 (95.0%)	1082 (98.4%)	1015 (96.1%)	2772 (97.3%)	2204 (95.5%)

The cohort experienced a relatively balanced number of fractures from skateboarding (55%, n = 2995) and snowboarding (45%, n = 2451) (Table 2). A higher percentage of women sustained snowboarding fractures (36% compared with 22% for skateboarding). Open fractures were more common among skateboarders (2%, n = 47) than snowboarders (0.2%, n = 6); overall, 53 out of 5446 fractures (1%) were open fractures (Table 2).

### High-Energy Trauma

High-energy trauma accounted for approximately 9% of skateboard-related fractures and 23% of snowboard-related fractures (Table 1). High-energy trauma incidents were more common among males, adults, and snowboarders (Supplementary Table 2). Associated fractures were more common in high-energy trauma regardless of the sport (7%) compared with low-energy trauma (3%).

### Seasonal Variation

**Figure 3.**
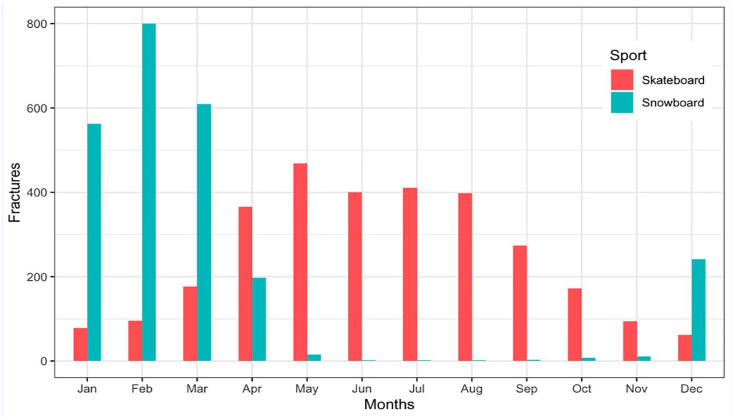
Bar plot illustrating the monthly variation among 5249 patients registered in the Swedish Fracture Register with a fracture resulting from skateboarding or snowboarding accidents, divided by sport.

A total of 98% of snowboarding fractures were sustained during December to April (Figure 3). The yearly fluctuation in skateboarding fractures was nearly the opposite, peaking during the spring and summer months. The curves intersect during the spring season, whereas the total number of fractures experiences a notable decrease in the autumn.

### Fracture Location

**Figure 4.**
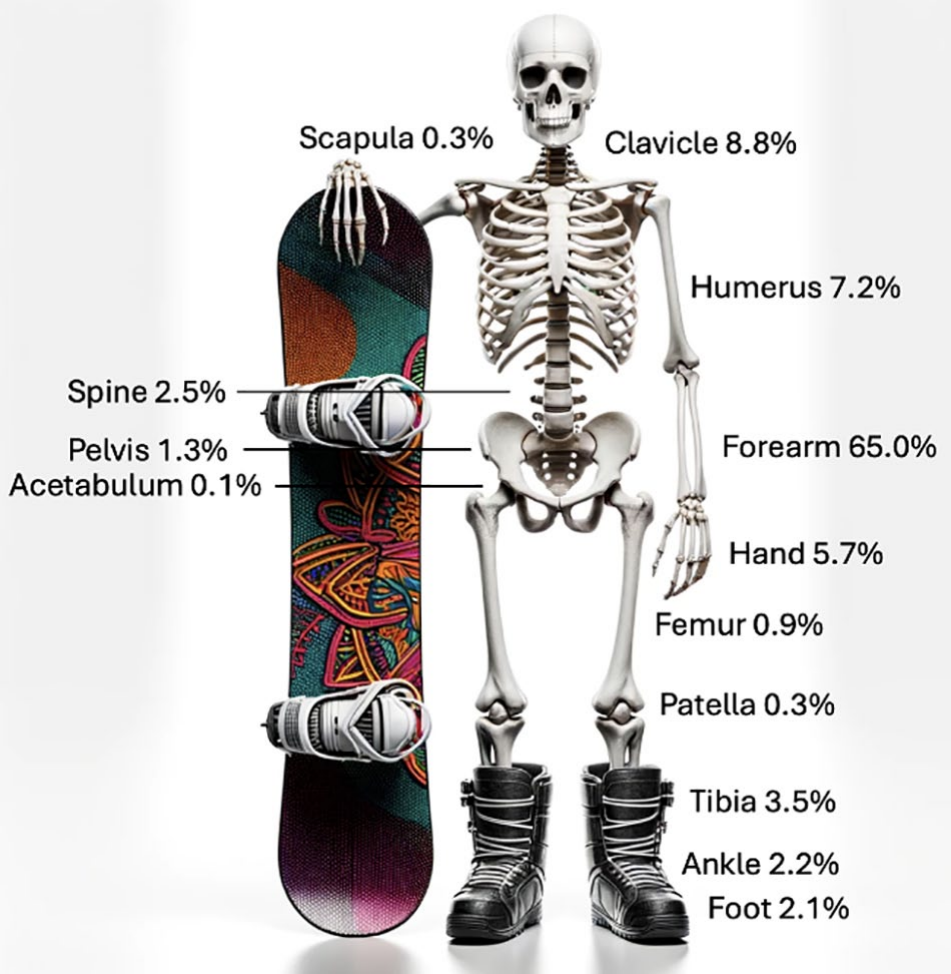
Fracture distribution for 2451 fractures registered in the Swedish Fracture Register after snowboarding accidents.

**Figure 5.**
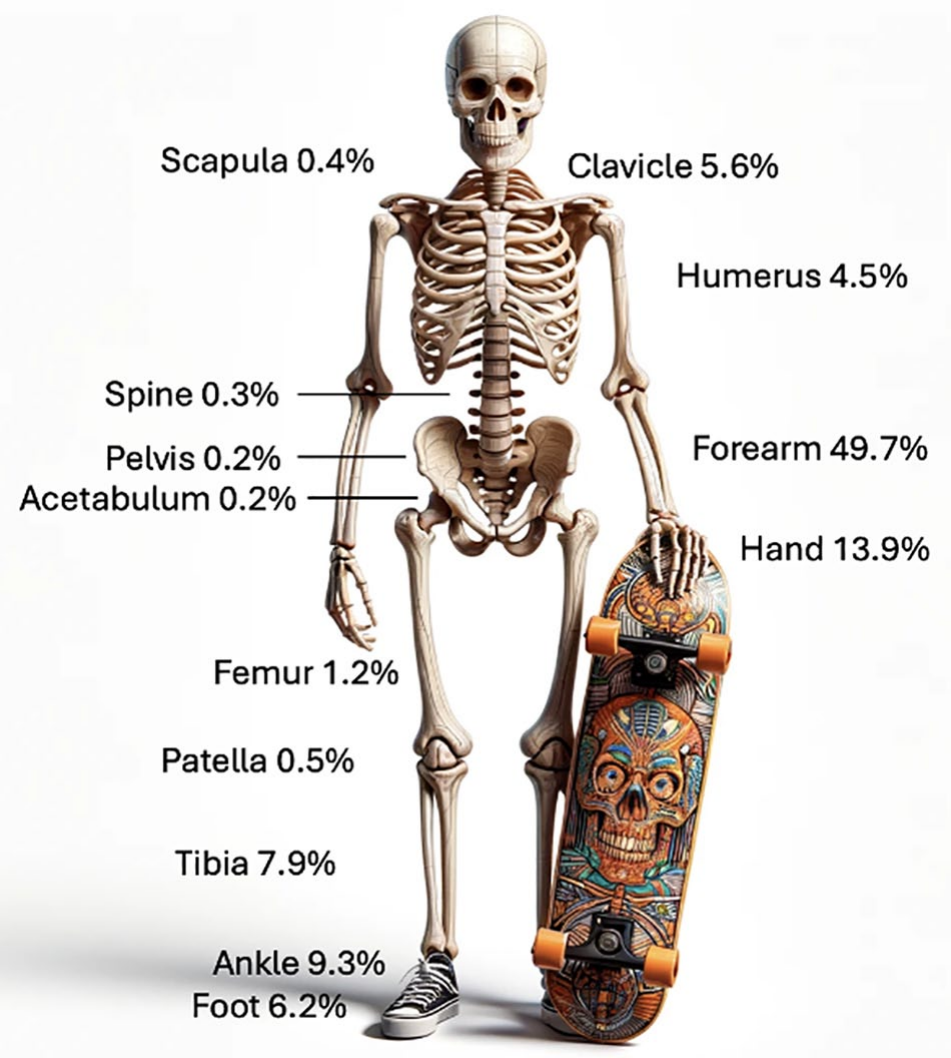
Fracture distribution for 2995 fractures registered in the Swedish Fracture Register after skateboarding accidents.

Upper extremity fractures represented the majority of injuries, accounting for 87% of snowboarding fractures and 74% of skateboarding fractures (Table 2, Figures 4 and 5). In both sports, forearm fractures were the most common. However, discrepancies were observed in lower extremity fractures. Among skateboarders, tibia fractures were twice as common, ankle fractures were four times more common, and foot fractures were three times more common compared with snowboarders.

**Table 2.** Fracture location (body part), open vs. closed fracture, and treatment for 5446 fractures sustained from snowboarding or skateboarding in the Swedish Fracture Register from January 2015 to December 2023. Distribution (number (%)). Patients aged ≥16 years at the time of injury were classified as adults

	Adult		Child		Overall	
	Skateboard (N = 1854)	Snowboard (N = 1342)	Skateboard (N = 1141)	Snowboard (N = 1109)	Skateboard (N = 2995)	Snowboard (N = 2451)
**Body part**						
Clavicle	130 (7.0%)	162 (12.1%)	39 (3.4%)	53 (4.8%)	169 (5.6%)	215 (8.8%)
Scapula	11 (0.6%)	8 (0.6%)	0 (0%)	0 (0%)	11 (0.4%)	8 (0.3%)
Humerus	65 (3.5%)	89 (6.6%)	70 (6.1%)	88 (7.9%)	135 (4.5%)	177 (7.2%)
Forearm	759 (40.9%)	716 (53.4%)	730 (64.0%)	877 (79.1%)	1489 (49.7%)	1593 (65.0%)
Hand	341 (18.4%)	114 (8.5%)	76 (6.7%)	26 (2.3%)	417 (13.9%)	140 (5.7%)
Spine	9 (0.5%)	59 (4.4%)	1 (0.1%)	2 (0.2%)	10 (0.3%)	61 (2.5%)
Pelvis	5 (0.3%)	27 (2.0%)	1 (0.1%)	5 (0.5%)	6 (0.2%)	32 (1.3%)
Acetabulum	4 (0.2%)	3 (0.2%)	1 (0.1%)	0 (0%)	5 (0.2%)	3 (0.1%)
Femur	24 (1.3%)	13 (1.0%)	13 (1.1%)	9 (0.8%)	37 (1.2%)	22 (0.9%)
Patella	9 (0.5%)	5 (0.4%)	6 (0.5%)	2 (0.2%)	15 (0.5%)	7 (0.3%)
Tibia	79 (4.3%)	50 (3.7%)	158 (13.8%)	36 (3.2%)	237 (7.9%)	86 (3.5%)
Ankle	269 (14.5%)	53 (3.9%)	10 (0.9%)	2 (0.2%)	279 (9.3%)	55 (2.2%)
Foot	149 (8.0%)	43 (3.2%)	36 (3.2%)	9 (0.8%)	185 (6.2%)	52 (2.1%)
**Open fracture**						
No	1825 (98.4%)	1338 (99.7%)	1123 (98.4%)	1107 (99.8%)	2948 (98.4%)	2445 (99.8%)
Yes	29 (1.6%)	4 (0.3%)	18 (1.6%)	2 (0.2%)	47 (1.6%)	6 (0.2%)
**Treatment**						
Nonoperative	1226 (66.1%)	904 (67.4%)	814 (71.3%)	911 (82.1%)	2040 (68.1%)	1815 (74.1%)
Operative	470 (25.4%)	293 (21.8%)	245 (21.5%)	127 (11.5%)	715 (23.9%)	420 (17.1%)
Missing	158 (8.5%)	145 (10.8%)	82 (7.2%)	71 (6.4%)	240 (8.0%)	216 (8.8%)

In snowboarders, 7 out of 18 talus fractures were classified as a “snowboarder’s fracture” (fracture of the lateral process of the talus^4^), whereas none of the 4 talus fractures in skateboarders were classified as such. “Skateboard elbow” is often used to describe fractures to the proximal forearm after repeated falls.^9,22^ Fractures to the proximal forearm was observed in 14.5% (n = 464) of skateboarding fractures but only 2.6% (n = 63) of snowboarding fractures. Fractures to the spine and pelvis were about 8 times more common in snowboarders: 93 spinal or pelvic fractures (3.8%) were registered in snowboarders compared with 16 (0.5%) in skateboarders (Table 2).

Comparing adult and pediatric cohorts, ankle fractures were 20 times more common and hand fractures were three times as prevalent in adults compared with children (Table 2). Conversely, forearm fractures were 50% more common in children. Among skateboarders, tibia fractures were three times more frequent in the pediatric population. Pediatric snowboarders sustained almost exclusively upper extremity fractures (94%).

### Treatment Modalities

Overall, 23% (n = 1135) of fractures were primarily treated operatively (Supplementary Table 3). Operative treatment was more common for open fractures (80%, n = 42) compared with closed fractures (22%, n = 1093), in adults (26%, n = 763) compared with children (18%, n =372), and in skateboarding fractures (26%, n = 715) compared with snowboarding fractures (19%, *n* = 420) (Table 3).

**Table 3.** Primary treatment for 5446 fractures sustained from snowboarding or skateboarding in the Swedish Fracture Register from January 2015 to December 2023 based on fracture location. Distribution (number (%)). Patients aged ≥16 years at fracture were classified as adults

	Nonoperative (N = 3855)	Operative (N = 1135)	Missing (N = 456)	Overall (N = 5446)
**Body part**				
Clavicle	294 (76.6%)	54 (14.1%)	36 (9.4%)	384
Scapula	12 (63.2%)	4 (21.1%)	3 (15.8%)	19
Humerus	202 (64.7%)	77 (24.7%)	33 (10.6%)	312
Forearm	2257 (73.2%)	587 (19.0%)	238 (7.7%)	3082
Hand	466 (83.7%)	53 (9.5%)	38 (6.8%)	557
Spine	64 (90.1%)	4 (5.6%)	3 (4.2%)	71
Pelvis	38 (100.0%)	0 (0.0%)	0 (0.0%)	38
Acetabulum	6 (75.0%)	2 (25.0%)	0 (0.0%)	8
Femur	4 (6.8%)	42 (71.2%)	13 (22.0%)	59
Patella	14 (63.6%)	6 (27.3%)	2 (9.1%)	22
Tibia	172 (53.3%)	114 (35.3%)	37 (11.5%)	323
Ankle	116 (34.7%)	176 (52.7%)	42 (12.6%)	334
Foot	210 (88.6%)	16 (6.8%)	11 (4.6%)	237
**Open fracture**				
No	3847 (71.3%)	1103 (20.5%)	443 (8.2%)	5393
Yes	8 (15.1%)	32 (60.4%)	13 (24.5%)	53
**Adult**				
Adult	2130 (66.6%)	763 (23.9%)	303 (9.5%)	3196
Child	1725 (50.0%)	372 (16.5%)	153 (6.8%)	2250
**Sport**				
Skateboard	2040 (68.1%)	715 (23.9%)	240 (8.0%)	2995
Snowboard	1815 (74.1%)	420 (17.1%)	216 (8.8%)	2451

For the entire cohort, lower extremity fractures were operated on in 39% of cases (n = 356), whereas upper extremity fractures were operated on in 19% (n = 775) (Table 3 and Supplementary Table 1). The body parts most likely to be treated operatively were the femur (91%, n = 42), ankle (60%, n = 176), and tibia (40%, n = 114). The distribution of specific characteristics per fracture location is presented in Supplementary Table 1.

## Discussion

### Demographics

The extent of overlap between populations practicing skateboarding and snowboarding is unclear. However, both sports primarily attract young males. As indicated in Figure 2, the peak age for participants in both sports is approximately 13 years old. Notably, a higher percentage of women sustained fractures while snowboarding compared with skateboarding, likely reflecting greater female participation in snowboarding. Previous reports indicate that both sports are male-dominated, with male participation at 75% for skateboarding and 61% for snowboarding.^6,23^ However, for children, the difference was smaller, indicating that there might be an ongoing shift toward higher female participation in skateboarding. Moreover, snowboarding fractures were more common in children, whereas skateboarding fractures were more prevalent in adults.

### Seasonal Variations

As expected, snowboarding injuries peaked during the winter months, whereas skateboarding injuries spiked during spring and summer. The seasonal variations overlap during the spring, and the lowest incidence of fractures was observed in late autumn (Figure 3). Due to Sweden’s extensive north-south span of over 1500 km, the arrival of spring varies significantly. While skateboarding can commence in southern Sweden during March, it remains the optimal season for snowboarding in the northern regions.

### Fracture Location

Upper extremity fractures constituted the majority of injuries in both sports, aligning with previous findings.^1,18^ Forearm fractures were the most prevalent injuries, suggesting similar fall mechanisms, such as falling onto an outstretched hand. However, significant variations were noted in lower extremity fractures. Fractures of the tibia, ankle, and foot were all more than twice as common in skateboarders.

This difference may be attributed to the use of protective boots and the secure attachment of snowboarders to their boards. However, a potential adverse consequence of locked boots is the possibility of generating distinct biomechanical loading that contributes to snowboarding-specific injuries, including the “snowboarder’s fracture,”^4,6^ which was not observed in skateboarders. The occurrence of spinal and pelvic fractures was infrequent in skateboarders but more common in snowboarders. One possible explanation is that street or park skateboarding is constrained by flat terrain and the rider’s push-off power, limiting speed and jump height. In contrast, snowboarders benefit from slopes and gravity, contributing to higher speeds and greater jump heights, increasing susceptibility to such injuries. However, the longboarding and vert subgroups within skateboarders can achieve speeds and heights comparable to snowboarders.^8^

Bissell et al^3^ found that snowboarders are more prone to fracture their left humerus. This finding is consistent with our results, where fractures of the humerus and clavicle were observed to be twice as common on the left side among snowboarders. One possible explanation is that the majority of snowboarders are regular-footed, leading with their left foot. Being attached to the board, regular-footed snowboarders may more frequently land on their left side during falls, increasing the risk of left-sided upper limb fractures. In contrast, skateboarders did not exhibit a preference regarding laterality, which could be due to their ability to dismount the board more freely and the variability in stance dynamics, resulting in a more even distribution of injuries between the left and right sides.

Comparing adult and pediatric cohorts, ankle fractures were more common in adults, whereas tibia fractures were more frequent in children, especially during skateboarding. According to SFR data, as patients age, there is a shift from sustaining tibia fractures to ankle fractures.^26^ Conversely, forearm fractures replace hand fractures in children, a pattern observed across other injury mechanisms.^26^

### Treatment Modalities

Skateboarders were more likely to be treated operatively, likely due to the increased occurrence of lower extremity fractures, which are commonly treated with surgical intervention. SFR data indicate a 29% operative treatment rate for upper extremity fractures compared with 70% for lower extremity fractures.^26^ In contrast, high-energy injuries were more than twice as common in snowboarders.

### Implications for Safety and Prevention

Reducing the frequency of injuries in boarding sports can be achieved by improving environmental conditions, providing safety education, and implementing protective equipment.^13^ Understanding fracture patterns is essential for developing better prevention measures. Snowboarders should emphasize protective gear for the wrists and upper extremities, in addition to helmet use common to both sports.^21^ Riders who engage in jumps and high-speed activities should consider using spinal protection. For skateboarders, protective gear for the upper and lower extremities is recommended.^22^ While securing the ankle might hinder the ability to propel the skateboard, designing specialized skateboarding footwear could be a potential solution. The distinctive requirement for specific elbow protection in skateboarding appears less critical in snowboarding, based on our data.

However, high-quality evidence on the effectiveness of protective gear is limited, as conducting randomized controlled trials in this area is challenging.

### Strengths and Limitations

The major strengths of this study include the large sample size, nationwide scope, and detailed fracture classification provided by the SFR. However, limitations exist. The inability to identify the overall incidence is a significant limitation due to the gradual implementation and varying completeness of the SFR. Even though there is 100% coverage for orthopaedic departments, fractures may be treated at primary care centers and, thus, would not be included in the SFR. Limited information on the injury location and circumstances (eg, designated ski slope, backcountry skiing, snow park, street, or skatepark) and the use of protective gear restricts a comprehensive understanding of injury mechanisms. In addition, the lack of a standardized definition for high-energy trauma in the SFR makes it reliant on the registering physician’s estimation. Examples are available upon registration; same-level falls are low energy, falls from height and traffic accidents high energy. Ultimately, it is up to the registering physician to determine. Moreover, we have no information on other associated injuries besides fractures. In pediatric fractures, there may be a tendency to record more fractures on long bones due to the presence of more comprehensive pediatric classifications in the SFR. As with all register-based studies, inherent limitations include miscoding, transfer errors, underreporting, and missing data.

## Conclusion

Although skateboarding and snowboarding share many similarities, particularly regarding the occurrence of upper extremity fractures, notable differences exist. Lower extremity fractures are more frequently encountered in skateboard accidents, whereas spine and pelvic fractures are more commonly seen in snowboarding. Specific injuries, such as the “snowboarder’s fracture” and “skateboard elbow,” are characteristic of each sport. Understanding these differences is crucial for developing targeted prevention strategies and improving safety measures for participants.

## Supplemental Material

sj-docx-1-sph-10.1177_19417381251353773 – Supplemental material for Fracture Epidemiology in Skateboarding vs. Snowboarding Supplemental material, sj-docx-1-sph-10.1177_19417381251353773 for Fracture Epidemiology in Skateboarding vs. Snowboarding by Viktor Schmidt, Mats Wadsten, Anders Brüggemann, Yasmin D Hailer and Olof Wolf in Sports Health

sj-docx-2-sph-10.1177_19417381251353773 – Supplemental material for Fracture Epidemiology in Skateboarding vs. Snowboarding Supplemental material, sj-docx-2-sph-10.1177_19417381251353773 for Fracture Epidemiology in Skateboarding vs. Snowboarding by Viktor Schmidt, Mats Wadsten, Anders Brüggemann, Yasmin D Hailer and Olof Wolf in Sports Health

sj-docx-3-sph-10.1177_19417381251353773 – Supplemental material for Fracture Epidemiology in Skateboarding vs. Snowboarding Supplemental material, sj-docx-3-sph-10.1177_19417381251353773 for Fracture Epidemiology in Skateboarding vs. Snowboarding by Viktor Schmidt, Mats Wadsten, Anders Brüggemann, Yasmin D Hailer and Olof Wolf in Sports Health

sj-tiff-4-sph-10.1177_19417381251353773 – Supplemental material for Fracture Epidemiology in Skateboarding vs. Snowboarding Supplemental material, sj-tiff-4-sph-10.1177_19417381251353773 for Fracture Epidemiology in Skateboarding vs. Snowboarding by Viktor Schmidt, Mats Wadsten, Anders Brüggemann, Yasmin D Hailer and Olof Wolf in Sports Health

## References

[1] Basques BA Gardner EC Samuel AM , et al. Injury patterns and risk factors for orthopaedic trauma from snowboarding and skiing: a national perspective. Knee Surg Sports Traumatol Arthrosc. 2018;26(7):1916-1926. 27177641 10.1007/s00167-016-4137-7

[2] Bergdahl C Nilsson F Wennergren D Ekholm C Möller M. Completeness in the Swedish Fracture Register and the Swedish National Patient Register: An assessment of humeral fracture registrations. Clin Epidemiol. 2021;13:325-333. 34045902 10.2147/CLEP.S307762 PMC8149280

[3] Bissell BT Johnson RJ Shafritz AB Chase DC Ettlinger CF. Epidemiology and risk factors of humerus fractures among skiers and snowboarders. Am J Sports Med. 2008;36(10):1880-1888. 18593842 10.1177/0363546508318195

[4] Boon AJ Smith J Zobitz ME Amrami KM. Snowboarder’s talus fracture: Mechanism of injury. Am J Sports Med. 2001;29(3):333-338. 11394605 10.1177/03635465010290031401

[5] Brudvik C. Child injuries in Bergen, Norway. Injury. 2000;31(10):761-767. 11154744 10.1016/s0020-1383(00)00093-0

[6] Corwin ZB Maruco T Williams N Romero-Morales M Rocha C Astiazaran C. Beyond the Board: Findings from the Field. https://pullias.usc.edu/wp-content/uploads/2023/10/Beyond-The-Board-Findings-From-The-Field.pdf

[7] Coury T Napoli AM Wilson M Daniels J Murray R Milzman D. Injury patterns in recreational alpine skiing and snowboarding at a mountainside clinic. Wilderness Environ Med. 2013;24(4):417-421. 24138836 10.1016/j.wem.2013.07.002

[8] Fabian LA Thygerson SM Merrill RM. Boarding Injuries: The Long and the Short of It. Emerg Med Int. 2014;924381:1-7. 10.1155/2014/924381 PMC3934587 24660063

[9] Forsman L Eriksson A. Skateboarding injuries of today. Br J Sports Med. 2001;35(5):325-328. 11579065 10.1136/bjsm.35.5.325 PMC1724407

[10] Hayes JR Groner JI. The increasing incidence of snowboard-related trauma. J Pediat Surg. 2008;43(5):928-930. 18485968 10.1016/j.jpedsurg.2007.12.041 PMC3731444

[11] Juto H Möller M Wennergren D Edin K Apelqvist I Morberg P. Substantial accuracy of fracture classification in the Swedish Fracture Register: Evaluation of AO/OTA-classification in 152 ankle fractures. Injury. 2016;47(11):2579-2583. 27645617 10.1016/j.injury.2016.05.028

[12] Kim S Endres NK Johnson RJ Ettlinger CF Shealy JE. Snowboarding injuries: Trends over time and comparisons with alpine skiing injuries. Am J Sports Med. 2012;40(4):770-776. 22268231 10.1177/0363546511433279

[13] Kirkpatrick DP Hunter RE Janes PC Mastrangelo J Nicholas RA. The snowboarder’s foot and ankle. Am J Sports Med. 1998;26(2):271-277. 9548123 10.1177/03635465980260021901

[14] Made C Elmqvist LG. A 10-year study of snowboard injuries in Lapland Sweden. Scand J Med Sci Sports. 2004;14(2):128-133. 15043635 10.1111/j.1600-0838.2003.00342.x

[15] Marsh JL Slongo TF Agel J , et al. Fracture and dislocation classification compendium - 2007: Orthopaedic Trauma Association classification, database and outcomes committee. J Orthop Trauma. 2007;21(10 Suppl):S1-S133. 10.1097/00005131-200711101-00001 18277234

[16] McKenzie LB Fletcher E Nelson NG Roberts KJ Klein EG. Epidemiology of skateboarding-related injuries sustained by children and adolescents 5-19 years of age and treated in US emergency departments: 1990 through 2008. Inj Epidemiol. 2016;3(1):10. 27747547 10.1186/s40621-016-0075-6 PMC4824795

[17] Möller M Wolf O Bergdahl C , et al. The Swedish Fracture Register—ten years of experience and 600,000 fractures collected in a National Quality Register. BMC Musculoskelet Disord. 2022;23(1):141. 35148730 10.1186/s12891-022-05062-w PMC8832767

[18] Quinn J Singh M Bennetto J Duong E Tudor F Platt S. The epidemiology of skateboarding injuries: A 10-year review at a major Australian centre. Cureus. 2024;16(3):e55624. 10.7759/cureus.55624 PMC10995764 38586808

[19] Rundgren J Bojan A Mellstrand Navarro C Enocson A. Epidemiology, classification, treatment and mortality of distal radius fractures in adults: an observational study of 23,394 fractures from the national Swedish fracture register. BMC Musculoskelet Disord. 2020;21(1):88. 32035488 10.1186/s12891-020-3097-8 PMC7007648

[20] Sachtleben TR. Snowboarding injuries. Curr Sports Med Rep. 2011;10(6):340-344. 22071394 10.1249/JSR.0b013e318237be2a

[21] Sadeghian H Nguyen B Huynh N Rouch J Lee SL Bazargan-Hejazi S. Factors influencing helmet use, head injury, and hospitalization among children involved in skateboarding and snowboarding accidents. Perm J. 2017;21:16-161. 10.7812/TPP/16-161 PMC5391782 28406787

[22] Shuman KM Meyers MC. Skateboarding injuries: An updated review. Phys Sportsmed. 2015;43(3):317-323. 26018674 10.1080/00913847.2015.1050953

[23] Ski Canada. Industry report - 2014/2015. https://www.skicanada.org/wp-content/uploads/2016/01/2014-15-Facts-and-Stats.pdf

[24] SLAO. Industry report - 2022/2023. https://www.slao.se/content/uploads/2023/07/SLAO-Branschrapport-22-23.pdf

[25] Stenroos A Handolin L. Incidence of recreational alpine skiing and snowboarding injuries: Six years experience in the largest ski resort in Finland. Scand J Surg. 2015;104(2):127-131. 24786173 10.1177/1457496914532249

[26] Swedish National Quality Registry for Fractures. Svenska Frakturregistret. https://sfr.registercentrum.se/in-english/the-swedish-fracture-register/p/HyEtC7VJ4

[27] von Elm E Altman DG Egger M , et al. The Strengthening the Reporting of Observational Studies in Epidemiology (STROBE) statement: Guidelines for reporting observational studies. J Clin Epidemiol. 2008;61(4):344-349. 18313558 10.1016/j.jclinepi.2007.11.008

[28] Wennergren D Stjernström S Möller M Sundfeldt M Ekholm C. Validity of humerus fracture classification in the Swedish fracture register. BMC Musculoskelet Disord. 2017;18(1):251. 28601085 10.1186/s12891-017-1612-3 PMC5466790

