# Supplementary material for: Fracture Epidemiology in Skateboarding vs. Snowboarding
Source: Sports Health. 2025 Jul 31;18(5):928–36. doi: 10.1177/19417381251353773 (PMC12316675; doi:10.1177/19417381251353773)
Supplement: Supplementary file 1 [file sj-docx-1-sph-10.1177_19417381251353773.docx]

| **Supplementary Table 1.** Specific characteristics according to fracture location (body part) of 5,446 fractures from snowboarding or skateboarding injuries in the Swedish Fracture Register from January 2015 to December 2023. Distribution (number (%)). Patients aged ≥16 years at the time of injury were classified as adults. | | | | | | | | | | | | | | |
| --- | --- | --- | --- | --- | --- | --- | --- | --- | --- | --- | --- | --- | --- | --- |
|  | **Acetabulum (N=8)** | **Pelvis (N=38)** | **Femur (N=59)** | **Foot (N=237)** | **Ankle (N=334)** | **Hand (N=557)** | **Humerus (N=312)** | **Clavicle (N=384)** | **Patella (N=22)** | **Spine (N=71)** | **Scapula (N=19)** | **Tibia (N=323)** | **Forearm (N=3082)** | **Overall (N=5446)** |
| **Open fracture** |  |  |  |  |  |  |  |  |  |  |  |  |  |  |
| No | 8  (100%) | 38 (100%) | 59 (100%) | 235 (99.2%) | 332 (99.4%) | 553 (99.3%) | 309 (99.0%) | 382 (99.5%) | 22 (100%) | 71 (100%) | 19 (100%) | 316 (97.8%) | 3049 (98.9%) | 5393 (99.0%) |
| Yes | 0  (0%) | 0  (0%) | 0  (0%) | 2  (0.8%) | 2  (0.6%) | 4  (0.7%) | 3  (1.0%) | 2  (0.5%) | 0  (0%) | 0  (0%) | 0  (0%) | 7  (2.2%) | 33 (1.1%) | 53 (1.0%) |
| **Treatment** |  |  |  |  |  |  |  |  |  |  |  |  |  |  |
| Non-operative | 6  (75.0%) | 38 (100%) | 4  (6.8%) | 210 (88.6%) | 116 (34.7%) | 466 (83.7%) | 202 (64.7%) | 294 (76.6%) | 14 (63.6%) | 64 (90.1%) | 12 (63.2%) | 172 (53.3%) | 2257 (73.2%) | 3855 (70.8%) |
| Operative | 2  (25.0%) | 0  (0%) | 42 (71.2%) | 16 (6.8%) | 176 (52.7%) | 53 (9.5%) | 77  (24.7%) | 54 (14.1%) | 6 (27.3%) | 4  (5.6%) | 4 (21.1%) | 114 (35.3%) | 587 (19.0%) | 1135 (20.8%) |
| Missing | 0  (0%) | 0  (0%) | 13 (22.0%) | 11 (4.6%) | 42 (12.6%) | 38 (6.8%) | 33  (10.6%) | 36 (9.4%) | 2  (9.1%) | 3  (4.2%) | 3 (15.8%) | 37 (11.5%) | 238 (7.7%) | 456 (8.4%) |
| **Adult** |  |  |  |  |  |  |  |  |  |  |  |  |  |  |
| Adult | 7 (87.5%) | 32 (84.2%) | 37 (62.7%) | 192 (81.0%) | 322 (96.4%) | 455 (81.7%) | 154 (49.4%) | 292 (76.0%) | 14 (63.6%) | 68 (95.8%) | 19 (100%) | 129 (39.9%) | 1475 (47.9%) | 3196 (58.7%) |
| Child | 1 (12.5%) | 6 (15.8%) | 22 (37.3%) | 45 (19.0%) | 12 (3.6%) | 102 (18.3%) | 158 (50.6%) | 92 (24.0%) | 8 (36.4%) | 3 (4.2%) | 0 (0%) | 194 (60.1%) | 1607 (52.1%) | 2250 (41.3%) |
| **Sport** |  |  |  |  |  |  |  |  |  |  |  |  |  |  |
| Skateboard | 5  (62.5%) | 6 (15.8%) | 37 (62.7%) | 185 (78.1%) | 279 (83.5%) | 417 (74.9%) | 135 (43.3%) | 169 (44.0%) | 15 (68.2%) | 10 (14.1%) | 11 (57.9%) | 237 (73.4%) | 1489 (48.3%) | 2995 (55.0%) |
| Snowboard | 3  (37.5%) | 32 (84.2%) | 22 (37.3%) | 52 (21.9%) | 55 (16.5%) | 140 (25.1%) | 177 (56.7%) | 215 (56.0%) | 7 (31.8%) | 61 (85.9%) | 8 (42.1%) | 86 (26.6%) | 1593 (51.7%) | 2451 (45.0%) |
