# Supplementary material for: Fracture Epidemiology in Skateboarding vs. Snowboarding
Source: Sports Health. 2025 Jul 31;18(5):928–36. doi: 10.1177/19417381251353773 (PMC12316675; doi:10.1177/19417381251353773)
Supplement: Supplementary file 3 [file sj-docx-3-sph-10.1177_19417381251353773.docx]

| **Supplementary Table 3.** Fracture treatment for 5,446 fractures sustained from snowboarding or skateboarding in the Swedish Fracture Register from January 2015 to December 2023. Distribution (number (%)). Patients aged ≥16 years at the time of injury were classified as adults. | | | | | | |
| --- | --- | --- | --- | --- | --- | --- |
|  | **Adult** | | **Child** | | **Overall** | |
|  | **Skateboard (N=1854)** | **Snowboard (N=1342)** | **Skateboard (N=1141)** | **Snowboard (N=1109)** | **Skateboard (N=2995)** | **Snowboard (N=2451)** |
| **Treatment** |  |  |  |  |  |  |
| Non-operative | 1227 (66.2%) | 906 (67.5%) | 871 (76.3%) | 926 (83.5%) | 2098 (70.1%) | 1832 (74.7%) |
| ORIF | 499 (26.9%) | 357 (26.6%) | 202 (17.7%) | 139 (12.5%) | 701 (23.4%) | 496 (20.2%) |
| External fixator | 5 (0.3%) | 1 (0.1%) | 0 (0%) | 1 (0.1%) | 5 (0.2%) | 2 (0.1%) |
| Arthroplasty | 5 (0.3%) | 0 (0%) | 0 (0%) | 0 (0%) | 5 (0.2%) | 0 (0%) |
| Other | 53 (2.9%) | 23 (1.7%) | 37 (3.2%) | 8 (0.7%) | 90 (3.0%) | 31 (1.3%) |
| Missing | 65 (3.5%) | 55 (4.1%) | 31 (2.7%) | 35 (3.2%) | 96 (3.2%) | 90 (3.7%) |
