# Supplementary material for: Fracture Epidemiology in Skateboarding vs. Snowboarding
Source: Sports Health. 2025 Jul 31:19417381251353773. Online ahead of print. doi: 10.1177/19417381251353773 (PMC12316675; doi:10.1177/19417381251353773)
Supplement: sj-docx-2-sph-10.1177_19417381251353773 – Supplemental material for Fracture Epidemiology in Skateboarding vs. Snowboarding [file sj-docx-2-sph-10.1177_19417381251353773.docx]

| **Supplementary Table 2.** Demographics and injury/fracture-related characteristics per energy level for 5,155 patients sustaining a fracture from snowboarding or skateboarding in the Swedish Fracture Register from January 2015 to December 2023. Mean (SD) and median [range] for age and distribution (number (%)) for other variables. Patients aged ≥16 years at the time of injury were classified as adults. | | | | | |
| --- | --- | --- | --- | --- | --- |
|  | **High-energy trauma (N=511)** | **Low-energy trauma (N=3466)** | **Missing (N=586)** | **Unknown  (N=592)** | **Overall (N=5155)** |
| **Age** |  |  |  |  |  |
| Mean (SD) | 22.9 (11.4) | 21.3 (11.4) | 21.2 (11.9) | 21.9 (12.0) | 21.6 (11.5) |
| Median [min, max] | 20.0 [5.00, 67.0] | 18.0 [1.00, 91.0] | 17.0 [5.00, 69.0] | 18.0 [4.00, 72.0] | 18.0 [1.00, 91.0] |
| **Sex** |  |  |  |  |  |
| Female | 118 (23.1%) | 1026 (29.6%) | 158 (27.0%) | 159 (26.9%) | 1461 (28.3%) |
| Male | 393 (76.9%) | 2440 (70.4%) | 428 (73.0%) | 433 (73.1%) | 3694 (71.7%) |
| **Adult** |  |  |  |  |  |
| Adult | 328 (64.2%) | 2003 (57.8%) | 319 (54.4%) | 349 (59.0%) | 2999 (58.2%) |
| Child | 183 (35.8%) | 1463 (42.2%) | 267 (45.6%) | 243 (41.0%) | 2156 (41.8%) |
| **Open Fracture** |  |  |  |  |  |
| No | 505 (98.8%) | 3436 (99.1%) | 582 (99.3%) | 585 (98.8%) | 5108 (99.1%) |
| Yes | 6 (1.2%) | 30 (0.9%) | 4 (0.7%) | 7 (1.2%) | 47 (0.9%) |
| **Associated** |  |  |  |  |  |
| At least 1 other fracture | 37 (7.2%) | 104 (3.0%) | 14 (2.4%) | 24 (4.1%) | 179 (3.5%) |
| None | 474 (92.8%) | 3362 (97.0%) | 572 (97.6%) | 568 (95.9%) | 4976 (96.5%) |
| **Sport** |  |  |  |  |  |
| Skateboard | 187 (36.6%) | 2061 (59.5%) | 338 (57.7%) | 262 (44.3%) | 2848 (55.2%) |
| Snowboard | 324 (63.4%) | 1405 (40.5%) | 248 (42.3%) | 330 (55.7%) | 2307 (44.8%) |
