# Supplementary figures and images for: Fracture Epidemiology in Skateboarding vs. Snowboarding
Source: Sports Health. 2025 Jul 31:19417381251353773. Online ahead of print. doi: 10.1177/19417381251353773 (PMC12316675; doi:10.1177/19417381251353773)

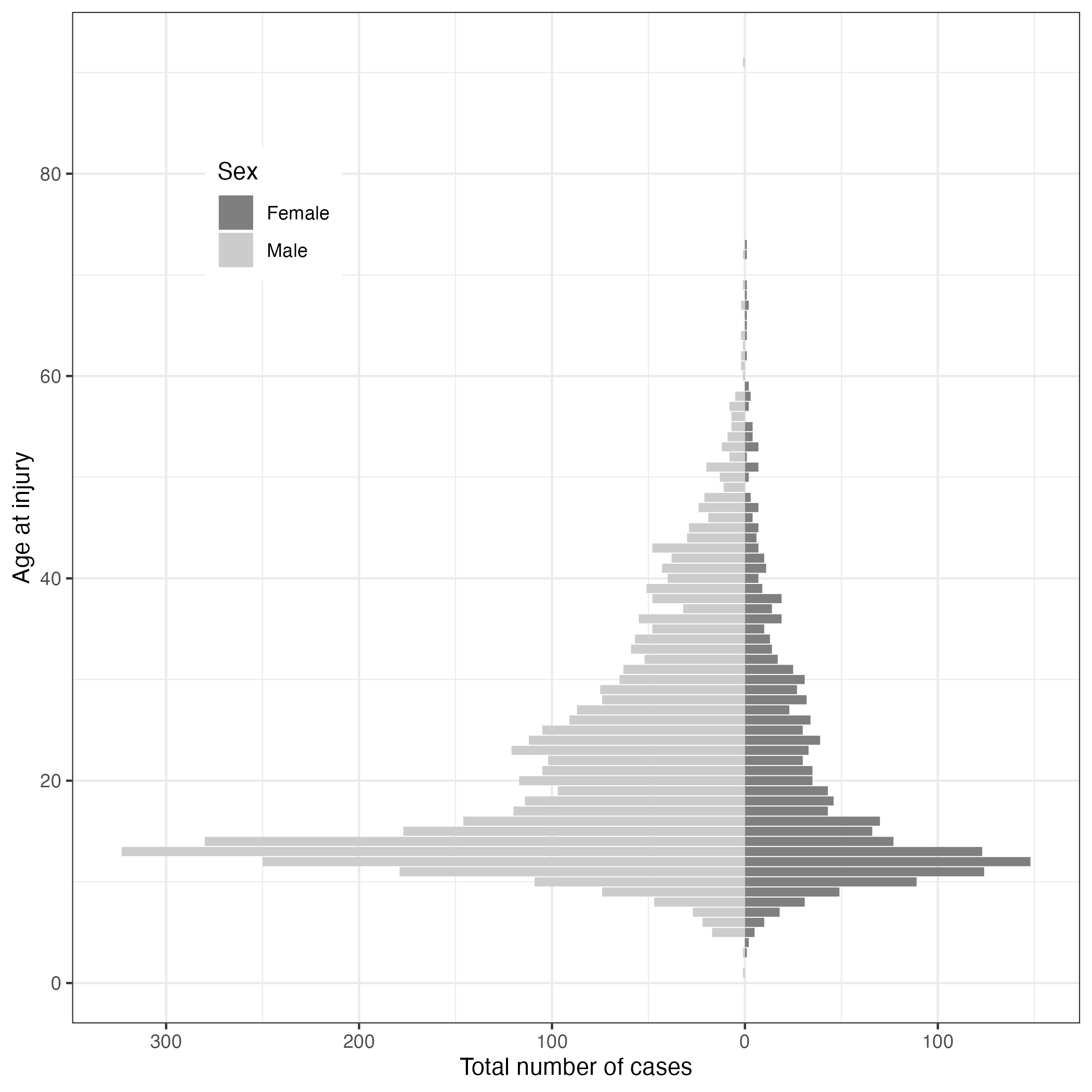

Supplement: sj-tiff-4-sph-10.1177_19417381251353773 – Supplemental material for Fracture Epidemiology in Skateboarding vs. Snowboarding [file sj-tiff-4-sph-10.1177_19417381251353773.tiff]
